# A New Method for Selective Extraction of Torularhodin from Red Yeast Using CO_2_-SFE Technique

**DOI:** 10.1007/s12010-024-04884-9

**Published:** 2024-02-22

**Authors:** Alfredo Ambrico, Vincenzo Larocca, Mario Trupo, Maria Martino, Rosaria Alessandra Magarelli, Anna Spagnoletta, Roberto Balducchi

**Affiliations:** Department for Sustainability, ENEA, Italian National Agency for New Technologies, Energy and Sustainable Economic Development, Trisaia Research Center, 75026 Rotondella, Italy

**Keywords:** Carotenoids, Red yeast, CO_2_-SFE, *Rhodotorula*, Torularhodin

## Abstract

Torularhodin is a dark pink colored carotenoid belonging to the xanthophylls group that can be biologically synthesized by red yeasts, especially by *Rhodotorula* and *Sporobolomyces* genera. The growing interest in this molecule is due to its biological activities such as antioxidant, anticholesterolemic, anti-inflammatory, antimicrobial, and anticancer. To satisfy potential commercial markets, numerous methods have been proposed to develop a cost-effective and environmentally friendly downstream process for the purification of torularhodin. However, obtaining high purity products without resorting to the use of toxic solvents, which can leave residues in the final preparations, remains a major challenge. In this context, the present study aimed to develop a new efficient method for the isolation of torularhodin from the red yeast *Rhodotorula* strain ELP2022 by applying the extraction technique with supercritical CO_2_ (CO_2_-SFE) in two sequential steps. In particular, in the first step, the dried lysed biomass of yeast was subjected to the action of CO_2_ in supercritical conditions (CO_2_SC) as sole solvent for extraction of apolar carotenoids. In the second step, the residual biomass was subjected to the action of CO_2_SC using ethanol as a polar co-solvent for the extraction of torularhodin. Both steps were carried out at different operating parameters of temperature (40 and 60 °C) and pressure (from 300 to 500 bar) with a constant CO_2_ flow of 6 L min^−1^. Regardless of the operating conditions used, this method allowed to obtain an orange-colored oily extract and a red-colored extract after the first and second step, respectively. In all trials, torularhodin represented no less than 95.2% ± 0.70 of the total carotenoids in the red extracts obtained from the second step. In particular, the best results were obtained by performing both steps at 40 °C and 300 bar, and the maximum percentage of torularhodin achieved was 97.9% ± 0.88. Since there are no data on the selective recovery of torularhodin from red yeast using the SFE technique, this study may be a good starting point to optimize and support the development of industrial production of torularhodin by microbial synthesis. This new method can significantly reduce the environmental impact of torularhodin recovery and can be considered an innovation for which an Italian patent application has been filed. In a circular bioeconomy approach, this method will be validated up to a pilot scale, culturing the strain *Rhodotorula* spp. ELP2022 on low-cost media derived from agri-food wastes.

**Graphical Abstract:**

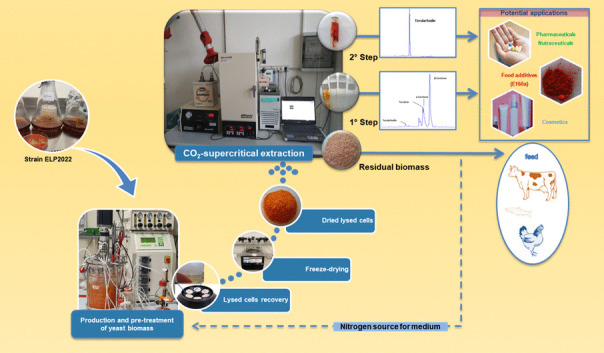

## Introduction

**Fig. 1:**
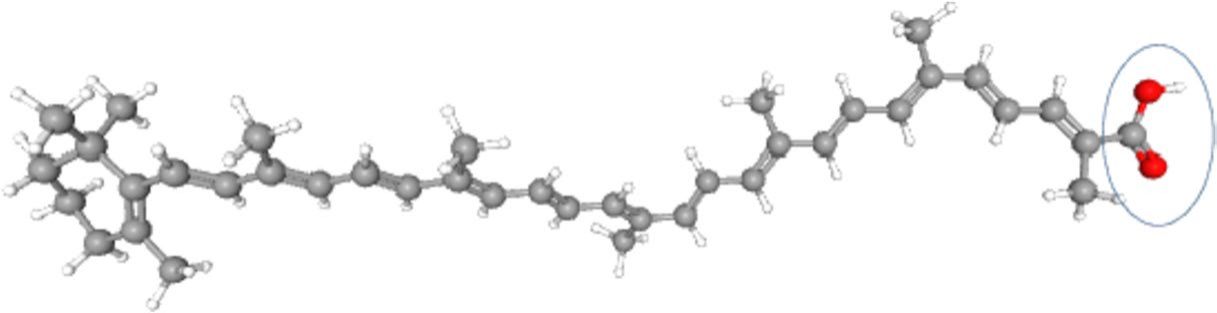
A 3D chemical structure of torularhodin with carboxyl group represented in the circle. Images obtained from PubChem (https://pubchem.ncbi.nlm.nih.gov/search/search.cgi)

Carotenoids are natural lipid-soluble tetraterpenoids generally classified into carotenes and xanthophylls [1]. The carotenes group has only carbon and hydrogen on his chemical structure, such as β-carotene, while xanthophylls, including torularhodin, contain, in addition, oxygen in their molecule [2]. Torularhodin is a dark pink-colored carotenoid that may be obtained by microbiological synthesis [3]. It has molecular formula of C_40_H_52_O_2_ with one β-ionone ring and presents a carboxyl group (Fig. 1) that makes it slightly more hydrophilic than carotenes [4].

Torularhodin is synthetized together with other carotenoids such as torulene, β-carotene, and γ-carotene by red yeasts belonging to the genera *Rhodotorula* (teleomorph is *Rhodosporidium*) and *Sporobolomyces* (teleomorph is *Sporidiobolus*) [5–7].

The first reports regarding the presence of torularhodin in the pigmented extracts of the *Rhodotorula* yeast biomass date back to the 1930s [8]. As early as 2002, it was reported that torularhodin has a more powerful effect on the scavenging of peroxyl radicals than even β-carotene [9]. However, nowadays, compared to other xanthophylls (especially astaxanthin), scientific information on torularhodin is still limited [3].

In recent years, there has been increasing progress in technologies and strategies to improve the production of torularhodin from microorganisms [10–12]. Torularhodin is considered a promising and valuable molecule due its biological activities such as antioxidant, anticholesterolemic, anti-inflammatory, antimicrobial, and anticancer proprieties [3].

Liu et al. [13] reported that torularhodin significantly scavenges free radicals and prevents oxidative damage in vitro and reduce D-galactose-induced hepatic oxidation. In the same year, a similar observation regarding the importance of multiple pathways in antioxidant damage of torularhodin-treated liver was also reported by Li et al. [14], while a more recent study reported that torularhodin effectively alleviated weight gain and insulin resistance in mice and may also lower blood lipids [15].

Sinha et al. [16] reported that yeast carotenoid extracts, including torularhodin, showed good efficacy on aggressive breast cancer cell lines. Similarly, Du et al. [17] suggest that torularhodin supplementation inhibits prostate cancer growth in nude mice by apoptosis of tumor cells.

Furthermore, torularhodin appears to possess interesting antimicrobial properties against gram positive and gram-negative bacteria [18, 19]. Even, Liu et al. [20] prepared torularhodin microspheres by electrospinning technology, and then their colonic targeting was studied by an in vitro intestinal simulation system. From their functional predictive results of intestinal microbiota indicated that torularhodin enhanced lysine biosynthesis of intestinal microbiota and reduced tyrosine metabolism.

In addition, the findings reported by Zhang et al. [21] suggest that torularhodin from *S. pararoseus* could offer a promising prevention strategy for neurodegenerative diseases as it effectively improved memory dysfunction, oxidative stress and neuroinflammation.

Despite its biological properties and health benefits, torularhodin is not yet industrially produced and is currently marketed only for research purposes at an average price of around 418 € per mg [22].

Nowadays, there is an increasing demand for red yeasts able to synthesize high quantities of torularhodin due to the important properties of this molecule [23].

In circular biorefining approach, the microorganisms are generally cultured on cost-effective fermentation medium utilizing secondary raw materials hydrolysate [16, 24, 25]. Ghiraldi et al. [26] used alperujo-based media as low-cost substrates to produce torularhodin and torulene in an integral approach for alperujo valorization. Also, Keskin et al. [27] reported that wastewater from oil mills, although possessing low in sugar and high in phenolic content, resulted in lower cell growth but increased carotenoid content and torularhodin could be selectively produced at high pH and with urea and glycerol supplementation.

Kot et al. [12] evaluated the use of waste glycerol fraction from biodiesel production and potato wastewater for carotenoids production by several *Rhodotorula* red yeast observing that carotenoids profile and content in biomass varied on basis of the yeast strain and culture medium composition.

The biosynthesis of torularhodin can be influenced by some exogenous factors (osmotic stress, white light irradiation, temperature, and oxidative stress) during yeast culture [28]. Grigore et al. [29] in a recent review synthetically highlighted that the production of torularhodin increases with higher temperatures and oxygen supply. Thumkasem et al. [30] reported lower temperatures favoring β-carotene synthesis in *Rhodotorula glutinis* and higher temperatures enhancing torularhodin and torulene synthesis.

Cardoso et al. [31] reported that torularhodin is the carotenoid synthesized in greatest quantity by *Sporobolomyces ruberrimus* when it is subjected to oxidative stress. Furthermore, it has been reported that biosynthesis can be enhanced by exposure to weak white light [32, 33].

Carotenoids, including torularhodin, are synthetized and accumulated in lipid micelles inside the cells, so several downstream operations are necessary to recover them effectively [24, 34]. This downstream process contributes significantly to production costs and include the following three phase: microbial cell wall disruption, extraction of carotenoids, separation, and purification of single carotenoids.

The cell disruption step is mandatory to extract intracellular molecules and can be achieved by mechanical, enzymatic and chemical methods [35, 36]. In general, the mechanical disruption techniques are the most widespread and traditionally include bead milling and sonication, although enzymatic and chemical methods are very interesting [37].

Subsequently to the cell lysis treatment, carotenoids need to be extracted from the yeast biomass, and conventional extraction procedures, based on the extraction capacities of different solvents, despite their different degrees of toxicity, are unequivocally the most universal ones adopted [38–40].

In general, based on the different polarity of carotenoids, dimethyl sulfoxide, acetone, methanol, ethanol, chloroform, hexane, petroleum ether, and/or their mixtures are mainly used [41–44].

However, new ecological approaches for the recovery of carotenoids from red yeasts are available in the scientific literature. Among them, the supercritical fluid extraction (SFE) technique has received particular attention for its extraction properties and capabilities compared to conventional methods.

In general terms, the supercritical fluid is any compound that has a temperature and pressure above the critical point where there is no distinct liquid and gaseous phase, so the properties of both forms are combined. These characteristics make supercritical fluids suitable as solvents for the extraction of compounds from biological matrices [45].

The most commonly used supercritical fluids for extraction purposes include water, methanol, ethanol, and carbon dioxide or mixtures thereof. Among these, supercritical carbon dioxide (CO_2_-SFE) requires much milder conditions, making it advantageous for the extraction of sensitive and thermolabile compounds such as carotenoids [46]. Indeed, CO_2_-SFE has been widely studied to recover carotenoids from various plant matrices and microalgae [47–50].

In few studies, the performance of supercritical carbon dioxide (CO_2_-SFE) for extraction of carotenoids from red yeasts has been evaluated [51–54].

Promising results were also reported in our recent study using CO_2_-SFE with and without co-solvent [55].

After extraction, regardless of the used methods, a mixture of carotenoids is generally recovered which, to obtain individual components with a high level of purity, as required in pharmaceutical and nutraceutical applications, needs further purification processes[56, 57].

The purification step can be achieved by several methods, such as chromatographic separation using synthetic resins as stationary phase. However, these methods involve the use of solvents which, although they perform well, in most cases, involve flammability risks and significant impacts on health and environment [58].

In the present study, based on the carotenoid profile we observed for a red yeast strain, we have developed and evaluated, at bench scale, a new method for the selective extraction of torularhodin using CO_2_-SFE. For this purpose, the biomass of a *Rhodotorula* strain (ELP2022) was produced in a 5-liter bioreactor and mechanically pretreated to lyse the cell wall before carrying out the extraction trials.

## Materials and Methods

### Yeast Strain

The *Rhodotorula* ELP2022 strain was isolated from a fresh cheese and identified at the microbiology laboratory of ENEA Research Centre Trisaia [55]. The pure culture was maintained on Potato Dextrose Agar (PDA, Sigma-Aldrich, Italy) at 4 °C and cryopreserved on 30% glycerol at − 80 °C.

### Production of Yeast Biomass by Sub-emerging Fermentation in Bioreactor, Mechanical Pre-treatment of Cells

The ELP2022 was grown on a mineral synthetic medium (MS medium) into 5-liter stirred bioreactor (B. Braun Biotech International, Germany) with initial working volume of 3 liters. The medium was composed of 5 g L^−1^ ammonium nitrate, 2 g L^−1^ yeast extract, 3 g L^−1^ dibasic sodium phosphate, 1.5 g L^−1^ potassium dihydrogen phosphate, 0.5 g L^−1^ magnesium sulphate, and 40 g L^−1^ of glucose. The bioreactor was inoculated with 10% (v/v) of microbial starter.

The latter was produced as follows: A loopful of yeast strain was picked up from glycerol stocks, streaked onto PDA plates and incubated at 26 °C for 120 h. After this time, two flasks (1 L Erlenmeyer baffled flask) containing 150 mL of YPD medium (10 g L^−1^ yeast extract, 20 g L^−1^ peptone, and 20 g L^−1^ glucose) were inoculated with single red colonies and placed into a thermostatic orbital shaker (Thermo Scientific Forma, model 420) for 96 h at 26 °C and 130 rpm.

During the process in bioreactor the pH, dissolved oxygen (pO_2_), foam production, stirrer speed, temperature, and air-flow rate were controlled by a Biostat® B unit (B. Braun Biotech International) equipped with a gas mixing module. The yeast culture was carried out at 28 °C, and pH was automatically maintained at 7.1 by the addition of 2 M sodium hydroxide or 2% sulfuric acid. The foam formation in bioreactor was controlled by the addition of the Anti-foam A (Sigma-Aldrich, Italy). PO_2_ was set to 40% of saturation by a cascade controller: first varying the rotation speed of the stirrer (min 120 rpm, max 240 rpm) than, after a delay time of 5 min, intervening with of a second cascade controller that introduced pulses of O_2_. Meanwhile, airflow was kept constant at 2.0 L min^−1^_._ Samples were taken at 24-h intervals to determine the dry cell weight (DCW) and to estimate the total carotenoids.

After 144 h of microbial growth, in order to disrupt the cells and improve the carotenoids extraction, the following mechanical pretreatment was performed. In brief, 4 liters of liquid culture were transferred into a sterile reactor chamber represented in a 10L PYREX^®^ bottle filled with 500 gr of glass beads (SiLibeads® type S, 1–1.3-mm diameter) and 33.6 g of NaHCO_3_. Then, the reactor chamber was shaken horizontally at 96 rpm overnight at room temperature. After this pretreatment step, the number of broken cells was determined by the Trypan Blue staining method, and the cell suspension was separated from the glass beads using a mesh sieve. Subsequently, the lysed cells were recovered by centrifugation at 9000 g for 10 min. The pellet was washed twice with water and dried using a freeze-dryer (Martin Christ, model Alpha 1–4). Dry biomass was weighed with an analytical balance (Kern, model 870) and stored at 4°C until used for SFE purposes.

### Determination of Total Carotenoids

To determine total carotenoids content, pretreated dried cells (0.2 g) were suspended in 10 mL of acetone/methanol mixture (7:3 v/v) as solvent and vortexed for 5 min. The suspension was centrifuged at 8900 g for 10 min, and the supernatant was removed and collected. This extraction process was repeated four times on the biomass.

The collected solvent (40 mL) was concentrated at 35 °C with a rotary evaporator (Steroglass Rotary Evaporator Instruments Kentron-Strike 202). After complete evaporation of the solvent, the weight of the extracts was determined, then resuspended in ethanol, and used directly to determine the absorbance at 453 nm by spectrophotometric analysis (Thermo Scientific – Multiscan GO) and the carotenoids profile by HPLC analysis.

The total carotenoids amount (μg) is estimated as follows:

$$TC\ \left(\mu g\right)=\frac{A\times V\times {10}^4}{E_{1 cm}^{1\%}}$$

where *A* is the absorbance, *V* is the total volume of sample solution (mL), and${E}_{1 cm}^{1\%}$ is the specific extinction coefficient of β-carotene for ethanol (2620 mL g^−1^ cm^−1^).

The carotenoid yield (CY) was expressed in terms of (μg/g) and is given in the following formula:

$$CY=\frac{TC}{W_{dc}}$$

where *TC* is the total carotenoids (μg) in the extract and *W*_dc_ is the weight (g) of dried cell.

### Determination of Carotenoids Profile by HPLC Analysis

To determine and quantify the percentage of different carotenoids present in the extract, an Agilent 1200 series HPLC system (Agilent Technologies) was used. The HPLC was equipped with degasser module (G1379B), binary pump (G1312B), auto-sampler (G1367B), column compartment (G1316A), UV-Vis (G1314B), and Diode Array (DAD) (G1315A) detectors. Before analysis, the extract was suspended in 1 mL ethanol with 0.2% (w/v) BHT and added into 2 mL vials through 0.22-μm syringe filters. The separation was performed on a Zorbax Rx-C18 analytical column (4.6 x 250 mm, 5 μm) eluting, in gradient mode, acetone, and water as reported by Ghilardi et al. [26]. The injection volume was 20 μL, and temperature was kept at 25 °C during the run. Direct UV absorption detection was performed at the characteristic wavelength for β-carotene (453 nm). Online spectra between 350 and 650 nm were recorded, and the single carotenoid identification was performed by comparing retention times (RT) and wavelengths for maximum absorbance (λ max) with literature data.

The concentration of main carotenoids was determined in terms of β-carotene equivalent (μg) for g of dried cell. For the purpose, synthetic β-carotene, purchased from Sigma-Aldrich (PHR1239-1G), was dissolved in hexane and diluted in ethanol with BHT to prepare standard stock solutions, and a calibration curve at the concentrations of 0.024, 0.012, 0.006, 0.0012, and 0.0006 [μg mL^−1^] was obtained. The analyses were carried out in three replicates, and data were collected using the software OpenLAB CDS Chemstation Edition Rev. C.01.10(201).

### Selective Recovery of Torularhodin from Lysed Biomass of Red Yeast Strain ELP 2022 by Application of Supercritical Carbon Dioxide

SFE-CO_2_ is an ideal isolation technique for separating hydrophobic or non-polar compounds such as carotenes. Meanwhile, polar compounds such as xantophylles group can be removed through the combination of SFE-CO_2_ and polar co-solvents [59].

Based on this principle and observing the carotenoid profile of red yeast ELP2022, in order to achieve the selective extraction of torularhodin, the lysed cell biomass was subjected to SFE-CO_2_ in two steps.

In the first step (S1), CO_2_ in supercritical conditions (CO_2_SC) was used as single solvent for extraction of apolar carotenoids such as torulene, γ, and β-carotene. In the second step (S2), the residual biomass was subjected to the action of CO_2_SC combined with ethanol as a polar co-solvent for the extraction of torularhodin.

The tests were carried out under different operating conditions that significantly influence the extraction efficiency and were carefully considered for efficient and selective recovery of carotenoids [60]. In particular, the effect of temperature (40–60 °C) and pressure (300–500 bar) was tested during step S1 maintaining a constant flow of CO_2_ (6 L min^−1^). Furthermore, in the step S2, the biomass previously subjected to the best operating parameters identified in the step S1 (400 bar, 40 °C), was subjected to CO_2_SC at different pressure and temperature conditions using anhydrous ethanol (≥ 99.5%) as co-solvent. The CO_2_ and co-solvent flows were 6 and 0.0005 L min^−1^, respectively. Both extraction steps lasted 60 min, and samples were collected into separate amber vials and stored at − 20°C until used for analysis.

**Fig. 2:**
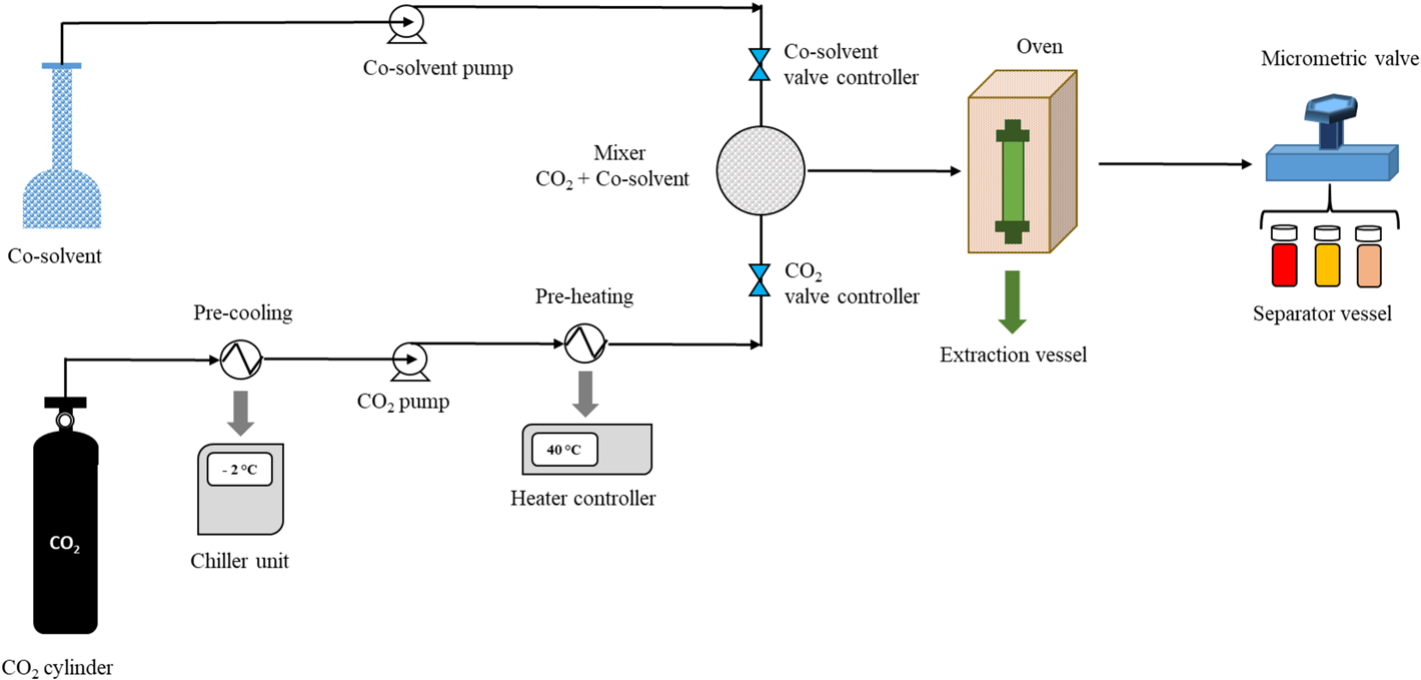
Schematic representation of the lab scale supercritical fluid extractor used for the two steps

The experimental conditions summarized in Table 1 were adopted in steps S1 and S2, respectively. The trials were performed using a bench-scale equipment (Applied Separations Spe-ed SFE-2, Allentown, PA) schematically shown in Fig. 2.

**Table 1:** Experimental plan in the first and second steps

Temperature (°C)	Pressure (bar)	
	1^st^ step: without cosolvent	2^nd^ step: with addition of ethanol at 0.5 mL min^−1^
40	300	300
	400	400
	500	500
60	300	300
	400	400
	500	500

For the trials, pretreated yeast biomass was micronized by a laboratory grinding mill, and approximately 1.5 g was loaded into a 50-mL extraction vessel whose volume were previously filled to 60% with glass microspheres (3 mm in diameter) to avoid biomass agglomeration and increase the contact surface between biomass and CO_2_.

### Statistical Analysis

Each experimental condition was investigated in triplicate. Each sample was analyzed thrice, and the averages of carotenoid yields, dry cell weight, and percentage area of the chromatogram peaks for major carotenoids were calculated. The means were separated by Tukey’s HSD test when the analysis of variance showed statistical significance (α = 0.05).

## Results and Discussion

### Production of Yeast, Mechanical Pre-treatment of Cells, and Total Carotenoids Determination

The aerobically culture of red yeast strain ELP2022 on MS medium performed in bioreactor has permitted to achieve 10.1 g L^−1^ of dry yeast biomass after 144 h, while the maximum quantity of total carotenoids of 293 μg g^−1^_(DCW)_ was recorded after 72 h.

**Fig. 3:**
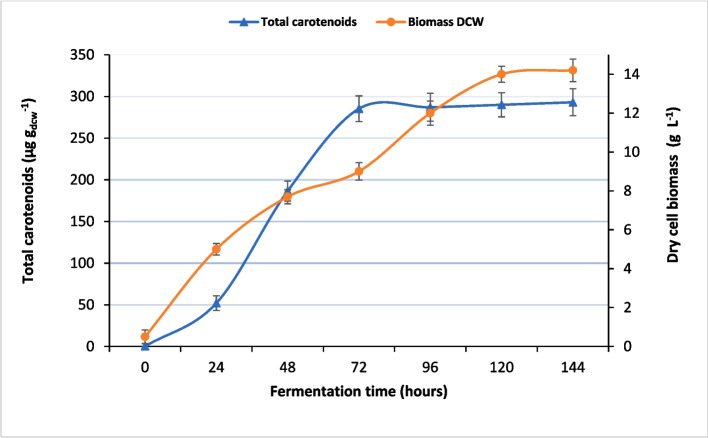
Time course of carotenoids and cell biomass production by *Rhodotorula* spp. (strain ELP2022) in 5L bioreactor

From Fig. 3, it is possible to see that carotenoid productivity was slower in the first 24 h of fermentation, corresponding to the lag phase of microbial growth. Subsequently, an exponential increase in the amount of produced carotenoids was recorded until the 72nd hour.

After this time, the increase in carotenoid content and cell mass became almost negligible.

These results agree with those of several authors, although the growth rate and carotenoid synthesis depend on the strain and culture conditions. For example, in a study by Sharma and Ghoshal 2020 [61] which aimed to optimize the production of carotenoids by *Rhodotorula mucilaginosa*, it is reported that the dry cell weight varies from 4.49 to 7.51 g L^−1^.

In particular, carotenoid yields similar to those obtained in this study were reported by Allahkarami et al. [62] for a *Rhodotorula* strain which, under optimal conditions, reached a maximum carotenoid content of 223.5 μg g_d.w_^−1^_._ While regarding microbial growth, our data are in line with those reported by Qi et al. [63], who, by cultivating a wild type of *Rhodosporidium toruloides* (ACCC20341) on tea waste hydrolysate, obtained 10.75 ± 0.65 g L^−1^ of dry cell biomass.

After mechanical pretreatment, the microscopic observation revealed that almost all cells appeared internally stained with Trypan Blue. This confirmed the effectiveness of the mechanical pretreatment performed by bead milling in 0.1 M sodium bicarbonate and agrees with our previous study and with the scientific literature [55, 64, 65].

### HPLC Determination of Carotenoids Profile in ELP2022

**Fig. 4:**
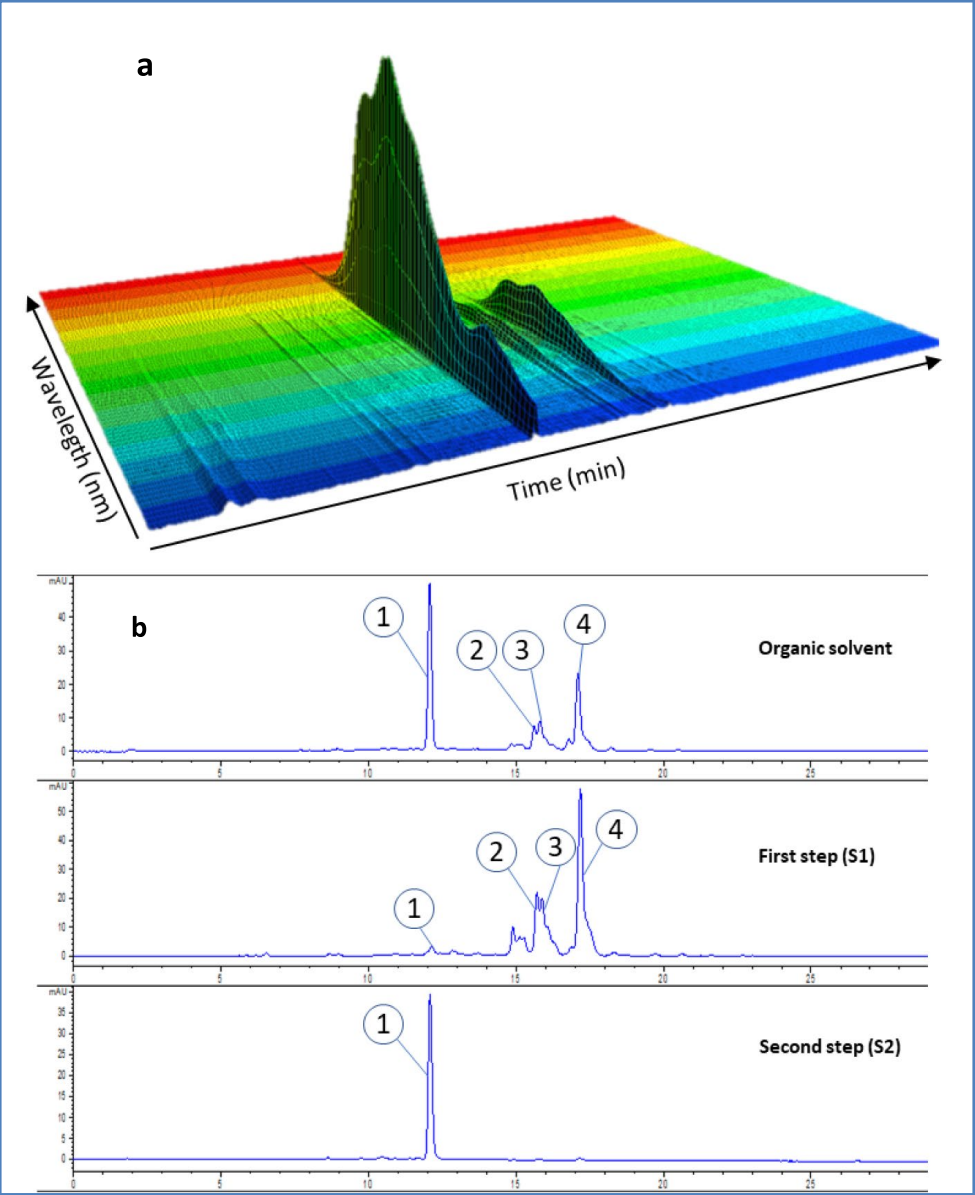
HPLC chromatograms at 453 nm for extracts obtained from strain ELP 2022 by organic solvent, SFE-CO_2_ first step S1 and SFE-CO_2_ second step S2. A 3D image showing spectral features and relative retention times for major peaks in extract by organic solvent extraction (a). The major peaks shown are as follows: 1, torularhodin; 2, torulene; 3, γ-carotene; 4, β-carotene (b)

The separation of carotenoids presents in organic solvent extracts obtained from *Rhodotorula* strain ELP2022 cultured in MS medium allowed to observe four main peaks (Fig. 4a).

Figure 4b shows a 3D spectrum image of the main peaks which were identified by their characteristic UV/Vis spectrum as torularhodin (retention time = 12.061 min; maximum adsorption at 498 nm), torulene (retention time=15.553 min; maximum adsorption at 483 nm), γ-carotene (retention time = 15.759 min; 462 nm), and β-carotene (retention time = 17.031 min, maximum adsorption at 453 nm). The peak related to β-carotene in the extract was further confirmed by the retention time observed for the peak of standard β-carotene.

Torularhodin was the main component and, due to its carboxyl group, had a shorter retention time than other carotenoids [6].

**Fig. 5:**
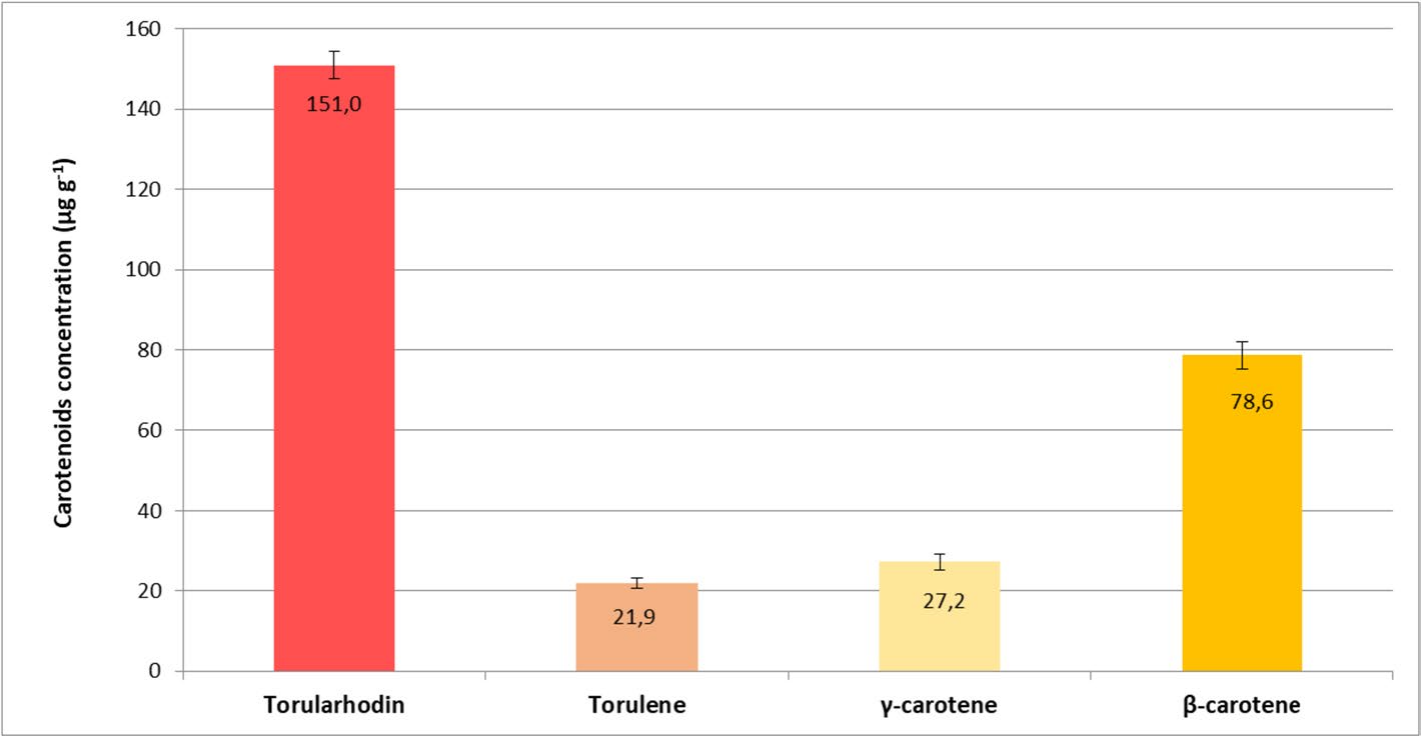
Concentration of major carotenoids present in dry cells of *Rhodotorula* spp. (strain ELP2022) extract by organic solvent method

As shown in Fig. 5, the amount of torularhodin was 151.8 μg g^−1^ and represented approximately 51.8% of the total carotenoids. β-carotene was the second component in terms of abundance with an amount of 78.7 μg g^−1^, while torulene and γ-carotene content was 22.2 and 27.5 μg g^−1^ respectively.

All together, they represent approximately 96% of the total carotenoids present in the extract.

Similar carotenoid profiles for yeasts belonging to the genus *Rhodotorula* have been reported in several independent studies.

In the work of Ungureanu et al. [66] where the authors cultivated the yeast *Rhodotorula rubra* ICCF 209 in bioreactor, on a medium very similar to the one we used, it was reported that the concentration of torularhodin represented 87% of the carotenoids in the mixture.

Clearly, the concentrations and the percentage ratios of the individual carotenoids can vary depending on the different metabolic synthesis of the particular species of *Rhodotorula* used and can be influenced by various factors, such as composition of the medium, pH, temperature, oxidative stress, during the culture [62, 67].

Elfeky et al. (2019) reported that carotenoid productivity for *Rhodotorula glutinis* could be enhanced under a high C/N ratio when ammonium sulphate is used as a nitrogen source [68].

### Selective Recovery of Torularhodin from Lysed Yeast Biomass by Application of Supercritical Carbon Dioxide

**Fig. 6:**
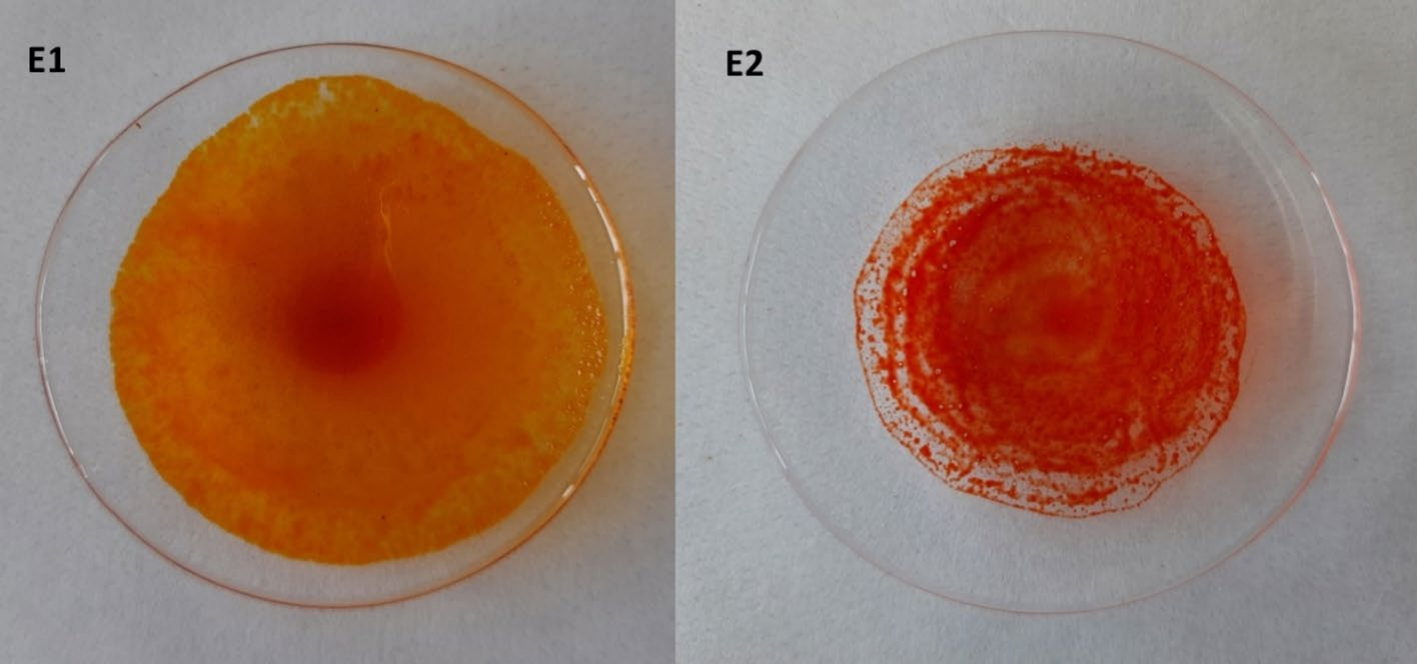
Extracts obtained from *Rhodotorula* spp. strain ELP2022 by SFE-CO_2_ performed in two steps. Extracts obtained after first step (E1) and second step (E2)

As shown in Fig. 6, the approach developed in this study for the selective extraction of torularhodin allowed to obtain an orange-colored oily extract (E1) and a red-colored extract (E2) after the first step and the second step, respectively.

As presented in Table 2, after the first step (S1), significant differences were observed in terms of carotenoid profile under different operating conditions. In particular, by comparing the data, it is possible to note that at 300 bar and 40 °C, the lowest quantity of torularhodin and the highest quantity of non-polar carotenoids in extract were obtained. Therefore, the trials of the second step (S2) were performed using the residual matrices obtained from the extractions in step S1 conducted using these operating conditions.

**Table 2:** Percentage of major carotenoids present in extracts obtained from lysed yeast biomass by SFE-CO_2_ (first step S1) at different operative conditions

		Area of peaks (%)			
Operative conditions		Torularhodin	Torulene	γ-Carotene	β-Carotene
40 °C	300 bar	4.3±0.25^**c**^	15.4±0.40^**a**^	23.5±0.55^**a**^	56.4±0.53^**a**^
	400 bar	6.3±0.21^**b**^	13.3±0.61^**c**^	21.7±1.76^**b**^	56.5±0.33^**a**^
	500 bar	7.5±0.31^**a**^	12.5±0.45^**d**^	20.4±0.49^**c**^	55.5±0.50^**b**^
60 °C	300 bar	6.5±0.32^**b**^	14.4±0.17^**b**^	19.2±0.38^**d**^	53.4±0.55^**d**^
	400 bar	6.5±0.36^**b**^	12.5±0.29^**cd**^	18.6±0.42^**d**^	52.0±0.14^**e**^
	500 bar	6.9±0.31^**b**^	14.7±0.40^**b**^	18.5±0.53^**d**^	54.4±0.29^**c**^

Area of peaks was determined at 453 nm. Data reported are mean value ± standard deviations. Different superscripts in the same column differ significantly (*P* < 0.05) according to Tukey’s test

The results presented in Table 3 clearly indicate that in the second step, an extract enriched with torularhodin can be obtained. Indeed, regardless of the operating conditions used during this step S2, torularhodin represented no less than 95% of the total carotenoids present in the red-stained extract. However, the best results were obtained at 40 °C and 300 bar where torularhodin achieved an average peak area percentage of 97.9±0.8.

**Table 3:** Percentage of major carotenoids present in extracts obtained from lysed yeast biomass by SFE-CO_2_ with co-solvent (Second step S2) at different operative conditions

		Area of peaks (%)			
Operative conditions		Torularhodin	Torulene	γ-Carotene	β-Carotene
40 °C	300 bar, EtOH 99.5%	97.9±0.88^**a**^	0.1±0.02^**c**^	0.1±0.01^**d**^	1.1±0.03^**d**^
	400 bar, EtOH 99.5%	95.2±0.70^**c**^	0.4±0.04^**a**^	0.9±0.08^**a**^	1.4±0.07^**c**^
	500 bar, EtOH 99.5%	96.2±1.08^**bc**^	0.1±0.05^**c**^	0.1±0.01^**d**^	1.1±0.05^**d**^
60 °C	300 bar, EtOH 99.5%	97.1±1.00^**ab**^	0.3±0.03^**b**^	0.4±0.02^**b**^	2.1±0.05^**a**^
	400 bar, EtOH 99.5%	96.5±0.40^**bc**^	0.2±0.01^**c**^	0.3±0.09^**c**^	1.8±0.02^**b**^
	500 bar, EtOH 99.5%	97.2±0.38^**ab**^	0.1±0.01^**c**^	0.2±0.01^**d**^	1.1±0.08^**d**^

Area of peaks was determined at 453 nm. Data reported are mean value ± standard deviations. Different superscripts in the same column differ significantly (*P* < 0.05) according to Tukey’s test

Torularhodin, as previously mentioned, contains carboxyl groups that can form hydrogen bonds with the intracellular system and CO_2_ alone as a solvent does not allow its exhaustive recovery.

It has been demonstrated that the extraction of intracellular carotenoids can be enhanced by particular compositions of mixed solvents. Mussaggy et al. [69], studying the sigma profile and sigma potential of the main carotenodes of *Rhodotorula glutinis* CCT-2186, concluded that torularhodin recovery is strongly influenced by the interactions between its hydrogen bonds with the system and those that it can form with the solvent. Indeed, in our tests the addition of a polar co-solvent such as ethanol allowed the recovery of torularhodin in the second step (S2).

The SFE method was applied to numerous matrices for the recovery of different substances including carotenoids [50, 59, 60, 70]. Approaches for the isolation of bioactive compounds using two-step SFE were also already studied and used.

For example, Sánchez-Camargo et al. [71] obtained extracts enriched in two phenolic compounds (carnosic acid and carnosol) from rosemary by a sequential supercritical fluid extraction in two steps that differ for the addition of a co-solvent. Vardanega et al. [72] proposed a two-step sequential SC-CO_2_ extraction process to selectively extract geranylgeraniol and tocotrienols from annatto seeds.

However, to the best of our knowledge, there are rare works focusing on the development of selective recovery processes of microbial carotenoids, especially astaxanthin, by SFE from red yeast.

In 2022, Lim et al. [73] started using of CO_2_-SFE to separate astaxanthin from the yeast *Phaffia rhodozyma*. In more recent studies, Harith et al. (2020) and Hasan et al. (2016) evaluated the efficacy of CO_2_-SFE to recovery the astaxanthin from enzymatically pre-treated cells of *Phaffia rhodozyma* and *Xanthophyllomyces dendrorhous*, respectively, while Martínez et al. [52] in 2020 have published a work regarding the extraction of carotenoids from the yeast *Rhodotorula glutinis* using CO_2_-SFE with and without the addition of co-solvent.

However, these authors did not obtain satisfactory results in terms of carotenoids recovery most likely due to the combination of techniques used in their study. Conversely, in our previous study, we recorded satisfactory recovery rate of total carotenoids ranging between 73.8% and 49.3% with and without co-solvent, respectively [55].

Anyway, these rare studies referred to the presence of torularhodin in a mixture with other carotenoids in extracts obtained by SFE-CO_2_ which required further purification processes. Indeed, the main techniques so far used for the extraction and separation of torularhodin from red yeasts are based on the use of solvents in combination with chromatographic techniques.

In a recent study, Zeng et al. [74], focusing on development of strategies to purify torularhodin after extraction from *Rhodotorula mucilaginosa,* used an elution solution composed of methanol/acetone/hexane (2/2/1, v/v/v) on a silica cartridge.

As previously mentioned, these techniques can lead to the production of potentially harmful and highly flammable wastes that are difficult to manage. Furthermore, solvents can residue in the extracts making the product not suitable for particular applications such, for example, those pharmaceutical. Indeed, solvent belonging to the class 1 and 2 should be respectively not present or limited in pharmaceuticals according to ICH guidelines Q3C(R8).

To date, selective extraction of torularhodin using a two-step SFE has not yet been applied and is reported for the first time in this study.

**Fig. 7:**
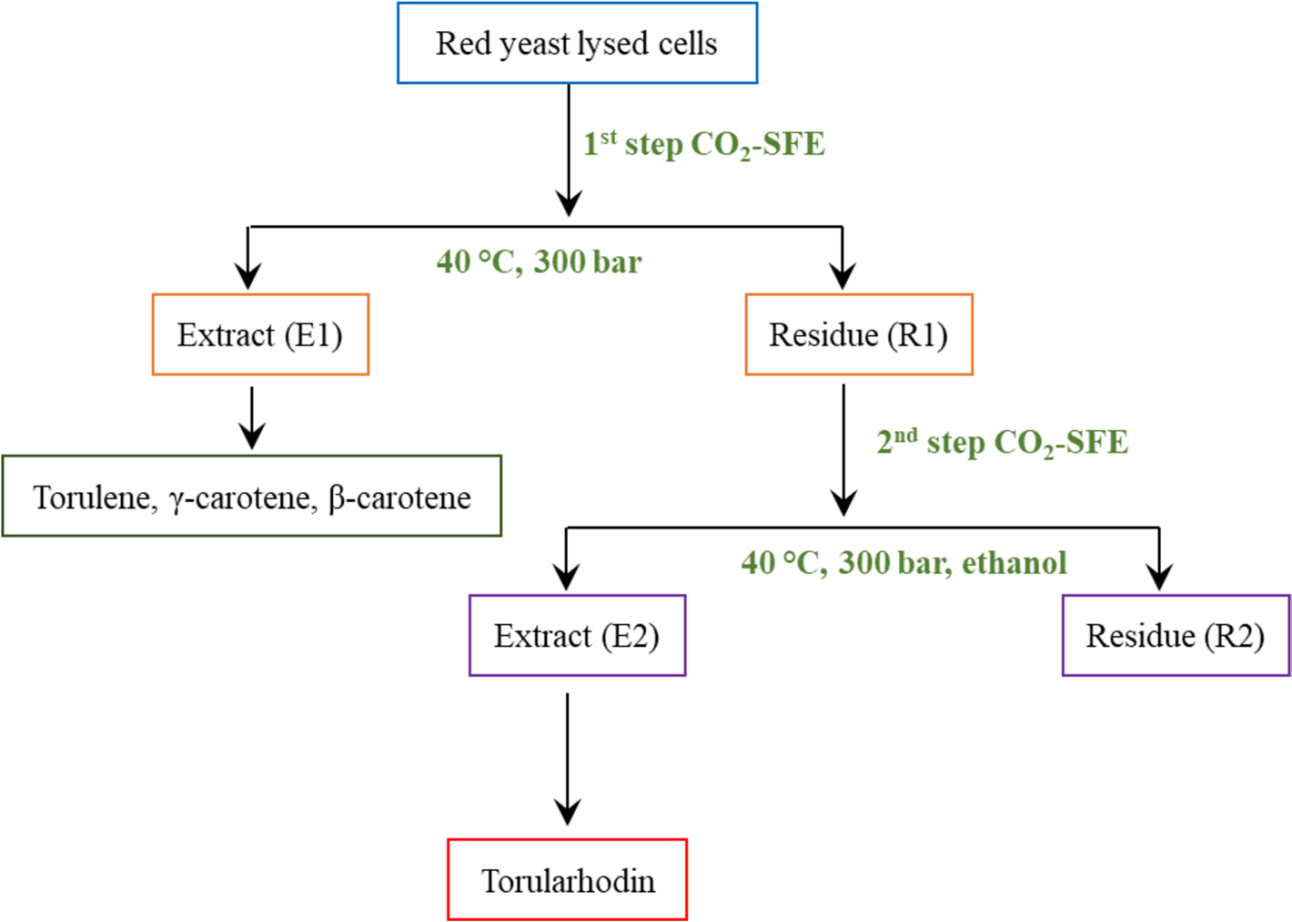
Schematic representation of the separation and purification of torularhodin from lysed biomass of *Rhodotorula* spp. strain ELP2022

In Fig. 7, it is presented the extraction scheme with the best performing conditions for both the sequential steps.

Compared to the conventional extraction method, it has been reported that CO_2_-SFE, in addition to being a simple, fast, efficient and environmentally friendly method, which does not cause environmental pollution, can preserve the biological activities of bioactive compounds in the extracts [75].

## Conclusions

In this work, the selective extraction of torularhodin from a red yeast belonging to the *Rhodotorula* genus (strain ELP2022) was carried out using CO_2_ under different supercritical conditions in two successive steps.

In the first step (S1), CO_2_ in supercritical conditions was used as the sole solvent, while in the second step (S2), the residual biomass was subjected to the action of CO_2_SC using ethanol as a polar co-solvent.

Considering the lack of data in the literature, our results represent an important starting point to optimize the selective extraction of torularhodin from red yeast by SFE and to achieve an acceptable extraction of this targeted molecule avoiding the co-extraction of other non-polar compounds that are recovered in the first step.

Indeed, due to the innovation in torularhodin extraction for this new method, an Italian patent application has been already filed with number 102023000018729.

This method can also be universally adopted for other red yeast strains capable of synthesizing torularhodin, although preliminary optimization may be necessary.

For the future, we plan to validate the developed method up to pilot scale by culturing the *Rhodotorula* spp. ELP2022 on a cheap medium derived from agri-food waste. Furthermore, it will be certainly interesting to assay in vitro and in vivo the biological activities of the extracts obtained by this method.

## Data Availability

Data generated during the current study will be made available from the corresponding authors on reasonable request.
